# Pulmonary Arteriovenous Malformations (PAVMs) and Pregnancy: A Rare Case of Hemothorax and Review of the Literature

**DOI:** 10.1155/2019/8165791

**Published:** 2019-04-28

**Authors:** Federica Di Guardo, Viviana Lo Presti, Giuliana Costanzo, Elisa Zambrotta, Luisa Maria Di Gregorio, Antonio Basile, Marco Palumbo

**Affiliations:** ^1^Department of Medical Surgical Specialties, University of Catania, Via Tindaro 2, 95124 Catania, Italy; ^2^Department of Medical and Surgical Sciences and Advanced Technologies ‘‘G.F. Ingrassia”, University of Catania, Via S.Sofia 78, 95123 Catania, Italy

## Abstract

Pulmonary arteriovenous malformations (PAVMs) are anatomical abnormalities consisting in a direct connection between pulmonary arteries and veins. Most of PAVMs are related to Hereditary Hemorrhagic Teleangiectasia, whereas only 10 to 20% are isolated sporadic cases. PAVMs tend to increase in size naturally; however, several factors can influence their growth such as pulmonary arterial hypertension, puberty, and pregnancy. Clinical manifestations are related to the right-to-left shunting and include dyspnoea, hypoxia, and pulmonary hypertension. The presence of PAVMs during pregnancy is associated with an increased risk of complications such as their rupture, haemothorax, and hypovolemic shock. The treatment reserved to PAVMs was the surgical resection of the lung lobe involving the malformation. Due to the worldwide acceptance of endovascular technique, the transcatheter embolization (TCE) is today considered as the mainstay of treatment. Recent studies reported the safeness of the TCE during pregnancy if performed by an experienced radiologist, at second or third trimesters when radiation exposure is believed to have minimal effect on the foetus. However, although the TCE during pregnancy represents an option, the treatment prior to pregnancy has to be considered the auspicial solution. Our study reports the case of a dyspnoeic pregnant woman with unknown pAVM causing hemothorax and simultaneously treated for pAVM reparation, left lower lobe resection, and hysterectomy. Postoperative treatment of embolization was performed to definitively close the pAVM.

## 1. Introduction

Pulmonary arteriovenous malformations (pAVMs) are rare congenital anomalies resulting from the direct communication between pulmonary arteries and pulmonary veins without the interposition of a capillary bed. Approximately 10 to 20% of patients with symptoms of pAVMs are isolated sporadic cases, whereas the remaining consistent percentage is related to Hereditary Hemorrhagic Teleangiectasia (HHT), an autosomal dominant vascular disorder also known as Osler–Weber–Rendu Syndrome [1, 2]. Other rare causes are represented by trauma, malignancy, hepatopulmonary syndrome, and cardiac surgery [3]. PAVMs tend to increase in size naturally but several factors influence their growing such as pulmonary arterial hypertension, puberty, and also pregnancy. Pregnancy has been considered as a precipitant factor for pAVMs; the reason is related to the increase of cardiac work and blood volume as well as the effect of progesterone on vessels during the gestation. In most of the cases, patients and pregnant women affected by pAVMs are asymptomatic, but when the clinical manifestations occur, they are often related to the right-to-left shunting and may include dyspnoea, hypoxia, and pulmonary hypertension [4]. Moreover, it has been reported that the presence of one or multiple pAVMs during pregnancy is associated with an increased risk of severe complications such as rupture, haemothorax, and hypovolemic shock [5]. Our study discusses a case of a symptomatic pregnant woman affected by a pAVM that caused a massive hemothorax compressing the correspondent lung. Moreover, it aims to review the previous literature about this topic focusing on symptoms, signs, diagnosis, and management of pAVMs in pregnant women, in order to avoid life-threatening complications.

## 2. Case Report

Our patient was a 32-year-old previously healthy female at the 39th week of gestation who accessed the first aim department of a primary healthcare centre of a peripheral hospital for severe dyspnoea and chest pain. Her past medical history did not present other hospitalizations for the same symptoms. Due to the clinical manifestations, the patient was initially treated as a case of pulmonary embolic disease and subjected to a massive anticoagulant therapy. Considering the clinical diagnosis and the child to term, an emergent caesarean delivery was performed in order to avoid foetal complications. The caesarean section was successfully performed under general anaesthesia using Stark's method due to the urgency related to the patient's clinical condition of increasing dyspnoea. Moreover, although the pAVM was still unknown at time of the caesarean section, the execution of spinal anaesthesia seems to be not indicated because of the risk of pAVM association with other AVMs, such as those located in the spinal cord, especially in case of HHT.

The foetal outcome showed an Apgar index of 3, 6, and 9, respectively, at minutes 1, 3, and 5; these data are in line with the administration of general anaesthesia and the acute maternal condition of severe dyspnoea.

Taking into account the foetal weight at birth, it showed a restriction of the expected value. The child weight was in fact 2590 gr at 39 weeks of gestation. However, ultrasounds performed during the pregnancy reported a reduction of the potential foetal growth from the 33 weeks of gestation without any Doppler alteration. This phenomenon should be the result of the chronical adaptation of the pregnancy to the unknown pAVM.

As far as the macroscopic exam of the placenta is concerned, a percentage of cotyledons infarcts inferior than 10% was reported.

Considering the patient's postoperative course, it showed a subsequently worsening of the clinical conditions, resulting in an acute distress syndrome that required an immediate transfer to the Gynaecology and Obstetrics unit of our structure. Due to the critical care panel and the low clinical conditions, the patient was intubated and housed in the ICU department. Considering the acute distress syndrome, a chest CAT scan was performed highlighting the presence of a left pAVM expanded, associated with a massive hemothorax that compressed the correspondent lung. The vital signs panel showed systolic blood pressure of 70 mmHg, diastolic blood pressure of 35 mmHg, pulse rate of 150/min, pulse oximetry saturation 88% on 100% inspired oxygen, afebrile temperature, and respiratory rate of 40/min. Initial labs revealed normal platelets, normal coagulation panel, and haemoglobin of 7 gm/dL. Critical care panel showed pH of 7.4, pCO2 of 43 mm hg, pAO2 60 mmHg, and saturation of 88%. After the placement of a chest tube, 3 litres of frank blood were removed; this action resulted in a normalization of the blood pressure and improved oxygenation on the monitor. The successive management was the clinical observation of the patient's conditions as well as the vital signs and labs test in order to perform the pAVM embolization when the patient clinical conditions will be stable. After three hours from the drainage, worsening of the patient conditions was observed reporting a severe collapse of the vital signs as well as a decrease of antithrombin III, fibrinogen, and haemoglobin values, with parameters of 33%, 122 mg/dl, and 5.8 gm/dl, respectively. Moreover, considering the postoperative caesarean course, the gynaecologic clinical evaluation showed a low uterine fundus contraction and the presence of conspicuous abnormal lochia. Uterine fundal massage was performed as first approach to solve the uterine low contraction followed by Credè's manoeuvre. Due to the failure of both, a pharmacological treatment was attempted starting with a simultaneous administration of intravenous Oxytocin (10-40 UI per 1 litre saline solution) and intramuscular Methylergometrine (0.2 mg one dose). The latter pharmacological approach involves the use of intravenous Sulprostone (0.5 mg per 1 litre saline solution) that was administered within half an hour from the signs of low uterine contraction and abnormal lochia. None of the previous pharmacological treatments succeeded.

**Figure 1:**
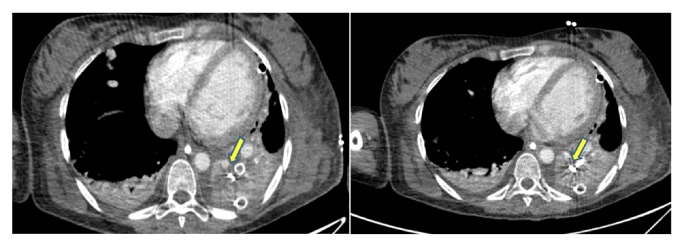
Chest CT showing signs of pAVM embolization and pulmonary resection of the LLL.

Considering the reproductive age of the patients, procedures as appositions of tamponade-balloon and embolization of the uterine arteries were taken into account but were not applicable in order of the unstable and precipitant parameters of the woman. Due to the patient's life-threating condition, a simultaneous surgical intervention of thoracic surgeons and gynaecologists had been necessary to solve the urgency, with the performance of a contemporaneous surgical reparation of the pAVM and resection of damaged left lower lobe (LLL) as well as a preventive hysterectomy to avoid the risk of disseminated intravascular coagulation (DIC). The surgical interventions were performed successfully but intraoperative blood transfusions and administration of antithrombin III and fibrinogen were necessary. The postoperative treatment showed a normalization of vital signs and labs panel as well as patient's clinical conditions. Due to the stable condition of the woman, the anaesthetist established the patient's autonomous breathing. After one week from the intervention, chest CT with intravenous contrast was performed showing a 4 cm area of active contrast. Pulmonary angiography confirmed the presence of a pAVM with feeding branch of a basilar left pulmonary artery supplying aneurysmal AVM and dilated draining vein. Transcatheter embolotherapy (TCE) of the culprit vessel was performed by placement of a nonadhesive liquid embolic agent (Onyx 34®). Repeated chest X-ray and chest CT after one week from TCE showed expansion of remaining left lung and signs of pAVM embolization and pulmonary resection of LLL, respectively (Figure 1). The patient course was subsequently uncomplicated and the discharging home happened after 14 days. MRI evaluation was performed in order to detect any head AVM but the result was negative. The genetic testing for HHT was not performed during this hospitalization period but the genetic examination performed a few months afterwards showed no association.

## 3. Discussion

PAVMs are uncommon congenital anomalies establishing a direct communication between pulmonary arteries and pulmonary veins. The most of cases of pAVMs are related to HHT, in which pulmonary AVMs develop in 15%–50% of patients [6], whereas only 10 to 20% of patients with symptoms of pAVMs are isolated sporadic cases. Pregnancy has been considered as a precipitant factor for pAVMs; the reason is related to the increasing of cardiac work and blood volume as well as the effect of the oestrogen-progesterone imbalance on vessels [4]. The patients are usually asymptomatic but the pAVMs right-to-left shunting is often the cause of several symptoms including hypoxia, pulmonary hypertension, and dyspnoea. Episodes of dyspnoea in pregnancy are not always directly related to an individual's health. In fact, dyspnoea should be the result of maternal adaptation to the gestation in terms of physiological changes of maternal respiratory function and gas exchange. It has been proven that at least 60-70% of pregnant women referred the sensation of air loss or more respiratory work. This sensation is more frequent starting from the 31st week of gestation. Anatomical change of the thoracic cage is one of the results of pregnancy. In addition, also maternal hormonal changes lead to modifications of the mucosa airways such as oedema and hyperaemia, as well as hypersecretion. On the other hand, dyspnoea in pregnant women is always to be taken into attention because of the several pathologies related to it, also the most uncommon such as pAVMs. As long as pAVMs complications are concerned, they can result in paradoxical septic or nonseptic emboli, brain, spinal, or systemic abscesses as well as secondary to septic emboli [7]. The existence of one or multiple untreated pAVM during pregnancy has been associated with a high risk of life threating complications with significant increasing of morbidity and mortality [8]; hence, detection and treatment of them should be recommended for asymptomatic pregnant women even if this approach is not accepted in all the countries [9]. The most scaring complications during pregnancy are represented by recanalization and rupture of pAVMs. The first one consists of the systemic supply to the malformation as response to the postprocedural local ischemia following the surgical or percutaneous treatment; this event should remain asymptomatic or manifest with recurrent haemoptysis. The second one is related to the increasing of the blood flow and cardiac output during the last two trimesters of pregnancy as well as the effect of the pregnancy hormonal imbalance on vessels leading to the sudden dilatation and eventually rupture of the pAVM. The consequences of this rupture develop into obstetrics emergencies such as haemothorax compressing the correspondent lung leading to the possibility of hypovolemic shock. Categories of patients requiring the screening for AVM have included patients affected by HHT with cerebral abscesses and young patients with an embolic cerebrovascular accident even if in presence of an apparent alternative cause [10]. PAVMS have always to be investigated in pregnant women with a diagnosis of HHT but the clinicians have to be suspicious of pAVMs also in case of pregnant women with pulmonary symptoms without any HHT evidence. Screening for pAVMs includes first-line procedures like arterial blood gas on oxygen and contrast echocardiogram to evaluate and eventually measure the right to left pulmonary shunting as well as a chest radiograph. While all these procedures are easy and fast methods, the limit is that they show a low sensitivity [11]. Highly sensitive screening tests include CAT scan (CT) and contrast echocardiography (CE) with more emphasis on CT that, easier than CE, permits a detailed anatomic detection, a precise location, and type definition of pAVM [12]. Scientific evidences consider the chest CT as the gold standard for pAVMs diagnosis, with the possibility to employ this procedure to schedule a subsequent embolization treatment and as follow-up [13]. The screening procedures are basically to be repeated every 5 years but in case of pregnancy may be scheduled due to the increased risk of pAVMs augmentation in number and size [9]. The treatment reserved to pAVMs was historically surgical resection of the anatomical-functional part of the lung involving the malformation. The endovascular technique of embolization has been recently accepted worldwide as mainstay of treatment and should be performed at time of diagnosis or when the following criteria are satisfied: progressive enlargement of a detected pAVM, paradoxical embolization, symptoms of hypoxemia and feeding vessels of 3 mm or larger [14]. A study about seven pregnant women with pAVMs treated with transcatheter embolotherapy (TCE) reported that this procedure is considered safe also when performed during pregnancy. In this case, some advices have to be considered: TCE should be performed by an experienced radiologist, preferably at second or third trimesters when the estimated radiation exposure is believed to have minimal effect on the foetus [15]. However, although the TCE during pregnancy represents a possible option, the treatment prior to pregnancy is always to be considered the auspicial solution.

## 4. Conclusions

Literature background showed several cases of pAVM, sporadic or associated with hereditary telangiectasia. Our study presented the case of dyspnoeic pregnant with unknown pAVM that expanded during pregnancy and caused hemothorax during the third trimester. The patient anamnesis did not report any previously significant medical history nor a diagnosis of Osler-Weber-Rendu syndrome. As discussed in our paper, the hemothorax represented an emergency that required a simultaneous approach of thoracic surgeons and gynaecologists performing, respectively, reparations of pAVM, resection of LLL and hysterectomy to avoid hypovolemic shock and CID. Postoperative treatment of embolization was performed to definitively close the pAVM. After our experience and a meticulous review of the recent literature, we suggest to preferably treat pAVMs prior to the pregnancy when the malformation is known. On the other hand, a second/third-trimester pregnancy with dyspnoea has always to be taken into attention and investigated due to the possible relation of this unspecific symptom with rare vascular anomalies such as pAVMs. In this last case, the possibility of severe complications such as hemothorax, hypovolemic shock, and CID are over the corner, so being suspicious of these rare vascular malformations in dyspnoeic pregnant women allows the clinicians to eventually use safe approach (TCE) to treat them, avoiding life threating complications.
